# Acute severe hypoxia induces prolonged cardiac transcriptomic remodeling despite rapid functional recovery

**DOI:** 10.21203/rs.3.rs-9603399/v1

**Published:** 2026-07-08

**Authors:** Steven Williams, Raymond Hendricks, Warren Burggren

**Affiliations:** The University of Texas Southwestern Medical Center; University of North Texas; University of North Texas

**Keywords:** Acute hypoxia, Cardiac function, Electrocardiography, Transcriptomic remodeling, Reoxygenation, Zebrafish

## Abstract

Electrocardiography and transcriptomic profiling were combined to quantify the short- and long-term effects of acute, severe hypoxia on adult zebrafish hearts. We hypothesized that rapid recovery of cardiac electrical activity does not necessarily indicate concurrent recovery at the molecular level. Continuous ECG recordings revealed pronounced bradycardia during hypoxic exposure, characterized by reduced heart rate and prolonged RR intervals. Upon reoxygenation, heart rate increased and RR intervals shortened within 2–5 min as oxygen levels normalized, MS-222 (tricaine) anesthesia was discontinued, and β-adrenergic stimulation (isoproterenol) restored cardiac excitability. Despite this swift physiological recovery, transcriptomic analyses conducted seven days post-hypoxic exposure demonstrated persistent differential gene expression in previously hypoxia-affected fish compared with normoxic shams. Upregulated genes were associated with stress response and tissue remodeling, whereas downregulated genes reflected cell-cycle regulation and metabolic adjustment. Together, these findings indicate a subtle transcriptional landscape consistent with cellular stabilization rather than ongoing injury. In summary, while ECG data alone might imply that acute hypoxic effects are transient, transcriptomic evidence reveals a sustained molecular imprint of hypoxic stress in the zebrafish heart that persists well beyond apparent functional recovery.

## Introduction

Reductions in oxygen availability in fishes typically elicit a pronounced bradycardia, reflecting adjustments in cardiac electrical activity that help stabilize circulation under low-oxygen conditions^1–5^. In zebrafish (*Danio rerio*), heart rate (*f*_*H*_) is governed by a sinoatrial pacemaker region and autonomic regulation, similar to other vertebrates^6–9^. As a result, changes in pacemaker discharge and conduction timing are readily captured by beat-to-beat variation in the RR interval on the electrocardiogram (ECG), making ECG-based analysis of *f*_*H*_ a powerful tool for assessing how hypoxia alters cardiac function and rhythm. Given these pronounced cardiac responses to hypoxia, zebrafish can be a valuable model for investigating how changes in oxygen level could alter cardiac electrophysiology in all vertebrates.

Despite the challenges associated with their comparatively small body size, a variety of approaches have been developed for recording cardiac electrical activity in adult zebrafish over the past two decades. While the classic in vivo surface ECG technique is commonly used to characterize conduction intervals and cardiac responses to pharmacological or environmental challenges^10–16^, subdermal needle-based single-lead ECG has not previously been applied in zebrafish. As with other single-lead ECG approaches, limitations in spatial resolution can restrict detailed waveform analysis and the reliable identification of chamber-specific arrhythmias. As such, several complementary techniques have been developed to improve ECG-based assessments by providing detailed information on cardiac activation patterns and pacemaker function. These approaches include in vitro isolated-heart preparations^17^, optical mapping of electrical activity^7,18^, and flexible microelectrode array recordings^19^.

Collectively, the methods outlined above, coupled with a growing baseline of how hypoxia affects zebrafish cardiac performance, positions zebrafish as a practical model for studying the cardiac conduction system function and cardiac rhythm control in vertebrates. Yet, a key knowledge gap exists—the absence of detailed, time-aligned ECG analyses that track how *f*_*H*_ and RR intervals change across periods of acute hypoxia exposure and the immediate post-hypoxic recovery (reoxygenation) in adult zebrafish. Previously, real-time ECG monitoring approach has been used in fetal sheep and some small mammals during experimentally induced hypoxia (e.g. *in utero* umbilical cord occlusion^20–22^). However, a similar experimental approach has not been applied to fishes using a controlled aquatic hypoxia–reoxygenation protocol. As a result, there is no quantitative, time-aligned description of how cardiac electrophysiological trajectories in adult zebrafish change during real-time acute hypoxia exposure and subsequent reoxygenation.

Beyond examining the functional (phenotypic) cardiac consequences of acute severe hypoxia via ECG, we also carried out a transcriptomic analysis of differential gene expression seven days into recovery. Few studies have investigated how the adult zebrafish heart responds at the molecular level once the acute hypoxia response has subsided and hypoxia-associated genes have returned to baseline (pre-hypoxia) expression levels. This post-hypoxia period is important because adult zebrafish undergo well-documented cellular and physiological recovery after hypoxic injury, including resolution of oxidative stress, clearance of apoptotic cells, and restoration of myocardial structure and function following hypoxia/reoxygenation^23^. Longer-term adjustments to oxygen stress can also reshape cardiovascular performance during hypoxia acclimation^24^, suggesting that additional downstream processes may continue to support cardiac homeostasis and tissue repair after the short-term hypoxia response has subsided. Yet, the gene-expression profile of hypoxia-exposed adult zebrafish hearts remains poorly defined with only a single study examining cardiac transcriptomic responses to hypoxia in zebrafish, focusing on chronic exposure^25^.

In this study, we used continuous, single-lead ECG to monitor cardiac electrical activity in lightly anesthetized adult zebrafish subjected to a controlled episode of severe acute hypoxia followed by rapid reoxygenation. We hypothesized that hypoxia would induce a pronounced bradycardia, reflected by prolonged RR intervals and reduced *f*_*H*_, that would be rapidly reversed upon reoxygenation. We further hypothesized that the reciprocal *f*_*H*_–RR relationship would remain intact but shift in accordance with hypoxic suppression and subsequent recovery. Although mathematically reciprocal to *f*_*H*_, analysis of RR intervals enabled quantification of dynamic changes in cardiac cycle duration across hypoxia and recovery. Next, we characterized the ventricular transcriptome seven days after hypoxic exposure, hypothesizing that acute hypoxic injury would produce a distinct and persistent transcriptional signature. Together, this combined electrocardiographic and transcriptomic approach was expected to provide an integrated view of how hypoxia and subsequent recovery shape both cardiac electrophysiology and gene expression in the adult zebrafish heart.

## Results

### Survival Outcomes During Hypoxia and Sham Procedures

A total of 30 fish were assigned to the hypoxia treatment group and 8 to the sham (normoxic) control group. Only 8 of the 30 (27%) hypoxia-exposed fish survived for > 24 h following the full hypoxia–reoxygenation protocol with most dying mid-procedure. Conversely, 7 of the 8 (88%) sham fish survived to complete the procedure. Accordingly, the reduced number of surviving hypoxia-exposed individuals resulted in a small sample size, which may have limited statistical power in some comparisons.

Most hypoxia-related mortalities occurred during either late in the severe hypoxia phase or during the early reoxygenation period. In these individuals, *f*_*H*_ never recovered to pre-hypoxia levels following reoxygenation, discontinuation of MS-222, and subsequent isoproterenol administration, and asystole typically occurred within ≤ 10 min of reoxygenation. Fish that failed to recover showed no resumption of independent opercular ventilation upon discontinuation of anesthesia, whereas hypoxia survivors exhibited a clear rebound in ventilation. It is possible that handling- and procedure-associated stress contributed to some early variability in *f*_*H*_, although these effects were minimized following the acclimation period and sham fish did not appear to be adversely affected. Diminishing R-wave amplitude was an additional indicator of impending asystole in some fish, however, this pattern was inconsistent across individuals and was therefore not used as a sole diagnostic criterion.

In contrast, sham fish recovered rapidly upon discontinuation of MS-222 anesthesia and isoproterenol administration. Opercular ventilation returned quickly (~ 1 min), and *f*_*H*_ rapidly returned to (and often exceeded) pre-reoxygenation values. Body movement of sham fish also returned sooner, evident from noticeable movement artifacts in the ECG traces within approximately 2–4 min after reoxygenation.

Few mortalities occurred during anesthesia induction or oral cannulation alone in either group, indicating that these steps (anesthetic dosage, cannula placement, electrode positioning) were not the primary source of mortality. Rather, the large disparity in survival reflects the physiological severity of the acute hypoxic exposure, which was intentionally designed to elicit strong and measurable cardiac depression.

### Electrophysiological Responses to Acute Hypoxia

ECG recordings revealed distinct patterns of cardiac activity across the hypoxia and sham protocols. Prior to hypoxia exposure, both groups showed a gradual decrease in *f*_*H*_, consistent with the known chronotropic depressive effects of MS-222^12^ (Fig. 2). As hypoxia progressed in the experimental group this reduction became more pronounced, reflected by longer RR intervals compared with the normoxic sham fish. Following reoxygenation, when MS-222 was removed and 10 μM isoproterenol was introduced, both groups exhibited a marked rebound in *f*_H_ characterized by shortened RR intervals. However, the sham group demonstrated a greater rate of increase in *f*_*H*_ compared to the hypoxia group during reoxygenation. By the + 5 min timepoint, both groups maintained an accelerated rhythm indicative of a sustained β-adrenergic response^7^ (isoproterenol).

Time-aligned LOESS analysis revealed distinct temporal patterns in cardiac rhythm across the − 15 to + 7 min interval relative to the time the fish began responding to reoxygenation (*t* = 0 min) (Fig. 3). During the baseline period (− 15 to − 10 min), both groups maintained similar *f*_*H*_ and RR interval values while declining at similar rates due to MS-222.

During hypoxia exposure (− 10 to 0 min), *f*_*H*_ progressively declined in both groups with a more pronounced reduction in hypoxia fish (Fig. 3a,b). In the hypoxia group, *f*_*H*_ decreased from approximately ~ 160 beats min^−^^1^ at − 10 min to ~ 130 beats min^−^^1^ at *t* = 0, while sham fish showed a more modest decline from ~ 160 beats min^−^^1^ to ~ 150 beats min^−^^1^ across the same interval. In line with this bradycardic response, RR intervals increased over the same period (Fig. 3c,d), rising from ~ 0.38 s to ~ 0.46 s in hypoxia fish and from ~ 0.38 s to ~ 0.40 s in sham fish. The lowest *f*_*H*_ and highest RR interval in hypoxia fish occurred near the end of the hypoxia period, immediately preceding reoxygenation onset. A slight pre-reoxygenation increase in *f*_*H*_ (~ − 2.5 min) was observed in LOESS-smoothed traces, but was not consistently present in the raw data.

Following reoxygenation onset (0 to + 7 min), cardiac recovery occurred progressively rather than instantaneously. In hypoxia fish, *f*_*H*_ increased from ~ 120 beats min^−^^1^ at *t* = 0 to ~ 140 beats min^−^^1^ by ~ + 2.5 min and continued rising to ~ 155–160 beats min^−^^1^ by + 7 min. Sham fish exhibited a more rapid and greater increase in *f*_*H*_, rising from ~ 150 beats min^−^^1^ at *t* = 0 to ~ 175–180 beats min^−^^1^ by ~ + 3–5 min, after which values fluctuated through + 7 min due to movement-related artifacts as sham fish recovered from anesthesia earlier than hypoxia fish. Concurrently, RR intervals decreased following reoxygenation, with hypoxia fish showing a gradual reduction from ~ 0.50 s at *t* = 0 to ~ 0.43 s by ~ + 2.5 min and further to ~ 0.38–0.37 s by + 7 min, whereas sham fish exhibited a more rapid shortening from ~ 0.40 s at *t* = 0 to ~ 0.34–0.33 s within the first few minutes of recovery and remaining within this range through + 7 min.

To formally evaluate the cardiac response to hypoxia and subsequent reoxygenation, LOESS-smoothed data were statistically analyzed across defined time windows (baseline: −15 to − 10 min; hypoxia: −10 to 0 min; recovery: 0 to + 7 min). For *f*_*H*_, two-way ANOVA revealed significant main effects of oxygen condition (hypoxia vs sham [normoxia]) (*p* < 0.0001) and time window (*p* < 0.0001), as well as a significant group x time interaction (*p* < 0.0001), indicating that *f*_*H*_ differed significantly between hypoxia and sham groups and also that these differences varied across the distinct time windows.

Analysis of RR interval revealed a similar but more pronounced pattern of divergence between the hypoxia and sham groups throughout the experiment. Two-way ANOVA identified significant effects of group and time window (*p* < 0.0001 for both), along with a significant group × time interaction (*p* < 0.0001), indicating a time-dependent separation between hypoxia and sham conditions. Post hoc Tukey comparisons showed that RR intervals were significantly longer in hypoxia-exposed fish during baseline (mean difference = 0.006 s, *p* < 0.001), although the magnitude of this difference was smaller than that observed in other comparisons. This difference increased during hypoxia exposure (mean difference = − 0.054 s, *p* < 0.001) and was further amplified during recovery (mean difference = − 0.081 s, *p* < 0.001), reflecting a sustained elevation in RR interval in hypoxia fish relative to sham controls.

Non-linear regression analysis revealed that *f*_*H*_ and the RR interval remained tightly coupled through a reciprocal relationship both before and after reoxygenation (Fig. 4). In the − 5 to 0 min window, the hypoxia and sham groups showed distinct curved relationships (Fig. 4a), each well-fitted to the model (R^2^ > 0.90). In the 0 to + 7 min window, both groups followed the same relationship but with shifted intercept (α) and scaling (b) parameter values (Fig. 4b).

Overlaying the curves from both windows demonstrated a consistent shift between the peak hypoxia and recovery intervals (Fig. 4c). Across both hypoxia and sham groups, the fitted *b* parameter increased from the − 5 to 0 min interval to the 0 to + 7 min interval (Fig. 4d). Differences between hypoxia and sham groups were also evident within each time window, indicating oxygen condition-specific shifts in the overall form of the *f*_*H*_–RR interval relationship while maintaining its underlying reciprocal structure.

### Transcriptomic Analysis

Transcriptomic analysis was performed on zebrafish ventricles harvested seven days following acute hypoxia exposure. Four biological replicates per condition were processed for RNA extraction, with each replicate comprised of 10 pooled ventricles from individual fish. One normoxia sample (N2_S2) was excluded from differential expression analysis due to sequencing failure, yielding only 28,888 total reads versus 5.9–17.9 million in the other seven samples. The remaining three normoxia and four hypoxia samples were used for downstream analysis.

In the sample-to-sample distance heatmap (Fig. 5a), three of four hypoxia replicates (H1, H2, H3) clustered with low intragroup distances. These three hypoxia replicates showed consistently greater distances to all normoxia samples than to each other. One hypoxia replicate (H4) clustered with normoxia samples rather than the hypoxia group, with lower distances to normoxia replicates than to the remaining hypoxia samples.

Normoxia replicates showed greater intragroup dispersion, consistent with the principal component analysis (PCA) results. PC1, accounted for 55% of total variance, while PC2 explained an additional 14% of variance. (Fig. 5b). Hypoxia replicates clustered tightly along both principal components, while normoxia replicates showed greater dispersion, particularly along PC1. Together, these analyses suggest that hypoxia induced a transcriptional response distinguishable from normoxic controls.

Differential gene expression (DEG) analysis revealed a small but distinct set of genes showing altered expression at seven days after acute hypoxic exposure. The minus average (MA) plot (Fig. 5c) shows the relationship between mean normalized gene expression levels and log_2_ fold change for all transcripts that were detected. Most genes clustered near a log_2_ fold change of zero, indicating broad transcriptomic normalization at seven days following hypoxic exposure. However, a discrete group of transcripts exhibited significant up– or down–regulation, highlighted in red and blue in (Fig. 5c), representing the molecular signature of cardiac tissue at seven days following hypoxic exposure. Lastly, the volcano plot (Fig. 5d) shows the direction and statistical strength of these changes.

To contextualize the gene-expression landscape identified at seven days post–hypoxia, differential expression results were compared to an adult zebrafish heart transcriptome following hypoxia^25^. To our knowledge, this remains the only available dataset examining hypoxia-mediated transcriptional changes in adult zebrafish heart tissue. Unlike our model, in which fish experienced a brief, severe hypoxic episode followed by immediate reoxygenation and seven days of recovery, Marques et al.^25^ exposed zebrafish to continuous hypoxia, with cardiac tissue collected during the hypoxic state. After remapping all microarray identifiers to current zebrafish gene symbols, only two genes overlapped between the datasets: *cyp1b1*, which was upregulated in both studies, and *btg2*, which was downregulated in both. No other shared DEGs were identified.

## Discussion

To examine cardiac function in fishes, many studies rely on non-ECG approaches such as *f*_*H*_ telemetry using implanted or external biologgers that detect mechanical heartbeats rather than electrical signals^26^, as well as Doppler flow probes or non-invasive electrode arrays^27,28^. Relatively few studies have recorded true ECGs in fishes, likely due to technical challenges. Where ECG has been used, applications have often been limited to *f*_*H*_ estimation or method development^27,29^. Among studies examining hypoxia, few have captured continuous cardiac electrical activity across both hypoxia and reoxygenation phases. Previous work in rainbow trout using implanted electrodes enabled monitoring during hypoxia but required invasive procedures and did not include controlled recovery, with mortality occurring prior to reoxygenation^30^. In contrast, the present approach enables continuous, time-resolved monitoring of cardiac electrical activity across both hypoxia and reoxygenation, providing a practical framework for examining dynamic cardiac responses to acute oxygen limitation in fishes.

Continuous ECG monitoring revealed that cardiac rhythm recovered rapidly following reoxygenation, with RR intervals shortening in parallel with increases in *f*_*H*_. Despite this rapid functional recovery, post-reoxygenation *f*_*H*_ remained lower in hypoxia-exposed fish compared to sham controls, indicating a transient limitation of cardiac performance following hypoxic depression. Because cessation of MS-222 and addition of isoproterenol coincided with reoxygenation, part of the observed increase in *f*_*H*_ reflects pharmacological effects in addition to restored oxygen availability. Comparison with sham fish provides an internal reference for this contribution, while the reduced recovery plateau in hypoxia-exposed fish suggests that intrinsic cardiac function remains temporarily constrained after hypoxic stress. Future studies separating oxygen restoration from adrenergic stimulation will be required to resolve their relative contributions.

Hypoxia-induced bradycardia is well documented across vertebrates, with teleost fishes exhibiting reductions in *f*_*H*_ under low oxygen conditions as an oxygen-conserving response^1,31^. Consistent with this response, *f*_*H*_ declined during hypoxia in adult zebrafish, accompanied by lengthening of RR intervals, indicating reduced pacemaker discharge frequency. Although the observed increase in RR interval (~ 0.05 s) may appear modest, it corresponded to an approximately 10% reduction in *f*_*H*_, highlighting the sensitivity of cardiac rate to small changes in cycle duration. Following reoxygenation, RR intervals shortened rapidly, paralleling recovery of *f*_*H*_. Notably, the reciprocal relationship between *f*_*H*_ and RR interval remained preserved across all experimental phases, indicating that hypoxia alters cardiac timing without disrupting the underlying pacemaker–cycle coupling. This suggests that modulation of heart rate during hypoxia and recovery occurs through adjustments in cycle duration rather than changes to the fundamental relationship governing cardiac rhythm.

To assess whether these physiological responses were accompanied by longer-term molecular changes, we examined the ventricular transcriptome seven days after hypoxic exposure. At this stage, gene expression reflected processes associated with injury recovery, including myocardial remodeling, extracellular matrix restructuring, immune signaling, and regulation of cellular stress pathways (Table 1.). This pattern is consistent with progression from acute hypoxia toward a longer-term repair and stabilization phase.

Comparison with the only available adult zebrafish cardiac hypoxia dataset (Marques et al.^25^) revealed minimal gene-level overlap. In that study, chronic hypoxia was associated with metabolic adjustment, proteostasis, and persistent HIF-associated signaling—features absent in our dataset. These differences indicate that the transcriptomic profile observed here represents a post-injury recovery state rather than an active hypoxia-response program.

Upregulated transcripts further support this interpretation. Elevated expression of *amfrb* suggests continued endoplasmic reticulum-associated degradation^32^ and ongoing resolution of proteostatic stress, while ECM-associated genes such as *serpinf1* indicate matrix remodeling and angiogenic signaling^33^. Increased *klhl41b* expression is consistent with maintenance of myofibrillar organization and sarcomeric integrity^34^, and elevated *nppb* suggests persistent wall-stress or endocrine signaling^35^. Collectively, these changes point to continued structural reinforcement and integration of stress-responsive pathways during late-stage recovery.

Conversely, downregulated transcripts indicate reduced cellular energy demand. Decreased expression of *nr1d4a* and *apoc1* suggests altered circadian-regulated metabolism and lipid turnover^36,37^, while reduced *btg2* expression reflects diminished antiproliferative signaling^38^. Downregulation of *rgs22* indicates altered GPCR-mediated signaling and coincides with normalization of cardiac function following hypoxia^39^. Together, these patterns define a transcriptional state consistent with cellular stabilization rather than ongoing hypoxic stress.

## Conclusion

Continuous ECG monitoring showed that cardiac rhythm recovered rapidly (~ 2–5 min) following reoxygenation, with the reciprocal *f*_*H*_–RR relationship remaining stable across all phases, indicating preservation of fundamental cardiac timing mechanisms under acute oxygen limitation. However, transcriptomic analysis revealed persistent molecular remodeling at seven days, characterized by longer-term structural, metabolic, and stress-related processes that differ from patterns observed during ongoing chronic hypoxia (Marques et al.^25^), supporting a post-injury repair state. Because this analysis assessed only a single time point at day seven of recovery, additional transcriptomic sampling across earlier windows (e.g. 24 and 72 h) would more fully resolve the temporal progression from acute hypoxia signaling to sustained post-injury remodeling. Together, these results highlight a dissociation between rapid functional recovery and persistent molecular remodeling, providing a framework for future studies of cardiac resilience and recovery across vertebrates.

## Materials and Methods

### Animal Husbandry

Adult wild-type zebrafish (*Danio rerio*, AB strain; ≥ 6 months old) were obtained from Ekkwill Waterlife Resources (Ruskin, FL, USA) and maintained in standalone recirculating aquarium systems at 27 ± 0.5°C, pH 7.2, salinity 0.5 ppt, and 100% oxygen saturation. Lighting was maintained on a 14:10 h light:dark cycle. Fish were fed 3 times daily with flake food in the morning and evening and frozen brine shrimp at noon. Water quality was tested twice weekly to monitor nitrogenous waste levels.

All procedures were approved by the University of North Texas Institutional Animal Care and Use Committee (IACUC Protocol #24012) and conducted in accordance with institutional guidelines.

### Acute Hypoxia Exposure Protocol

Adult zebrafish were anesthetized via submersion in system aquarium water (0.5 ppt salinity, 27 ± 0.5°C, pH 7.2) containing 75–100 mg·L^−^^1^ (0.0075%–0.01%) buffered MS-222 (Sigma Aldrich Inc., St. Louis, MO, USA) for 2 min. MS-222 has depressive effects on *f*_*H*_^12^, so this conservative level of anesthesia was used to minimize its effect on experimental results. Also, the duration of the ECG procedure was necessarily short because survival during severe hypoxia exposure is low and prolonged anesthesia exacerbates this risk. However, just as excess anesthetic would have further lowered *f*_*H*_, insufficient anesthesia would have failed to suppress spontaneous movements, producing motion artifacts and complicating electrode placement. After accounting for these variables, zebrafish were carefully transferred onto the hypoxia exposure apparatus and positioned dorsally for the ECG procedure once anesthesia induced equilibrium loss and cessation of gill ventilation.

Because of the technical demands of the ECG recording procedure, a custom-built apparatus was created. Anesthetized fish were positioned dorsally atop a flexible, water-impermeable fabric that conformed to a U-shaped trench (width = 1.5 cm, length = 5 cm, depth = 1.5 cm) carved into a rigid rectangular foam insert (thickness = 9 cm) wedged securely inside a 10 cm × 16 cm acrylic box housing. The foam insert was positioned at a slight tilt, directing the opercular outflow of water away from the fish’s body (opposite the cannula side) where it drained through a tubing at the end of the trench, returning system water to the reservoir for recirculation. Importantly, the shallow angle of the trench bed also allowed the slow opercular outflow to briefly pool around the fish’s dorsum before draining, ensuring that approximately one-third of the fish’s body height remained submerged for the duration of the procedure. This prevented desiccation of the fish’s body while also keeping the ventrally positioned ECG microneedle electrodes dry.

The recirculating system water was stored in two separate 1 L reservoirs, each equipped with single 3 watt, 50 GPH mini submersible pump. Outflow tubing from both pumps (flexible PVC, 4.8-mm inner diameter) converged at a three-way stopcock, with connections between the tubing and stopcock made using Luer fittings. Both pumps operated continuously, enabling rapid switching between reservoirs during the procedure. The first reservoir contained 100 mg·L^−^^1^ MS-222 as the maintenance anesthetic dose and was supplied with both compressed air and nitrogen gas through separate tubing lines, each delivering gas via its own air stone positioned at the base of the reservoir. Adjusting the flow rate of either gas to this reservoir allowed for precise control of dissolved oxygen level, where compressed air was used to produce normoxic water (pO_2_ = 21 kPa) and nitrogen gas produced hypoxic water (pO_2_ = 2–3 kPa). A second reservoir was continuously aerated with compressed air, ensuring that the water remained normoxic, and was never treated with nitrogen gas. The normoxic water in this reservoir was supplemented with 10 μM isoproterenol (used in place of epinephrine because of its greater stability in solution) to provide β-adrenergic support during recovery^7^, contained no MS-222, and was used exclusively during the reoxygenation and recovery phase of the experiment. The dissolved oxygen levels in both reservoirs were monitored using a benchtop DO meter (Hanna Instruments, HI 5421–01, Woonsocket, Rhode Island, USA). Lastly, the water parameters in both reservoirs including temperature (27 ± 0.5°C), salinity (0.5 ppt), and pH (7.2) were identical to the system aquarium water parameters in which the fish were originally housed to prevent physiological disturbances unrelated to changing dissolved oxygen levels.

To prevent atmospheric oxygen from dissolving into the recirculating system water during the hypoxia exposure stage, the open-air top of the acrylic box housing containing the trench bed and fish was sealed with a gasketed lid. Additionally, nitrogen gas was delivered via flexible PVC tubing to the bottom of the acrylic housing (2 L min^−^^1^ beneath the foam insert) through a port drilled near the base. Nitrogen introduced at the bottom displaced ambient air upward and exited through the small opening in the gasketed lid that was used for the passage of the ECG electrodes. Finally, the mouth of the reservoir was partially covered with sealing film (Parafilm^®^) to allow outflow of excess nitrogen gas, which in turn prevented the inflow of atmospheric oxygen. During both the normoxic acclimation and reoxygenation phases, the gasketed lid and film-sealed coverings were removed to allow atmospheric oxygen to dissolve into the system water to enhance reoxygenation.

To begin the procedure, a short (2 cm) section of PE-190 polyethylene tubing (1.19-mm internal diameter) fitted over a blunted 18-gauge, 38-mm (1.5-inch) length needle was inserted orally, acting as a buccal cannula that continuously supplied water from either reservoir A or B to the fish throughout the procedure. The 18-gauge needle hub was connected to the three-way stopcock plumbed in-line with the reservoir pumps, allowing rapid switching between reservoirs and precise control of water flow rate. The flow rate was adjusted using the stopcock and was maintained between 25–40 mL·min^−^^1^. The cannula assembly was secured to a post connected to a micromanipulator for precise control during cannula insertion. Cannulation required gently opening the fish’s mouth (avoiding piercing tissue) with a fine-hooked instrument while carefully advancing the cannula using the micromanipulator knob to prevent injuring the fish by excessive insertion depth. Routinely, the insertion depth of the cannula tip into the mouth ranged only from 3 to 5 mm depending on the size of the fish. Once cannulated, ECG needle electrodes were inserted (see below) and fish were allowed to acclimate for 5 during ECG monitoring while receiving normoxic system water with anesthetic.

After acclimation, the hypoxia exposure phase was initiated by first shutting off the compressed air supply oxygenating the water in the first reservoir, then turning on the nitrogen gas to this reservoir to initiate the conversion of water from normoxic to hypoxic. During this period, water flow to the fish remained uninterrupted. The small reservoir volume (1 L) coupled with the flow of nitrogen gas allowed for the water to rapidly transition from normoxic to hypoxic in ~ 1 min. Fish remained under hypoxia for 10 min prior to reoxygenation.

Reoxygenation was initiated by cutting off the hypoxic water supply from the first reservoir and switching the inflow to the second reservoir, which contained pre-aerated normoxic water supplemented with 10 μM isoproterenol. At this time, the gasketed chamber lid was also removed to allow atmospheric oxygen to re-enter the system. Fish remained under reoxygenation conditions until spontaneous gill ventilation resumed (~ 10 min) as the light anesthesia faded and isoproterenol supplementation increased *f*_*H*_, at which point movement artifacts prevented further usable ECG recording. After this reoxygenation period, fish were transferred to 3 L tanks for further observation and recovery under their original husbandry conditions.

### Single-Lead Electrocardiography (ECG)

Cardiac electrical activity was recorded using a single-lead ECG method adapted from a previously described approach^13^. Three 29-gauge, 12-mm stainless-steel needle electrodes (MLA1213, ADInstruments, Colorado Springs, CO, USA) were mounted on independent posts secured to micromanipulators for precise positioning and adjustment.

**Figure 1:**
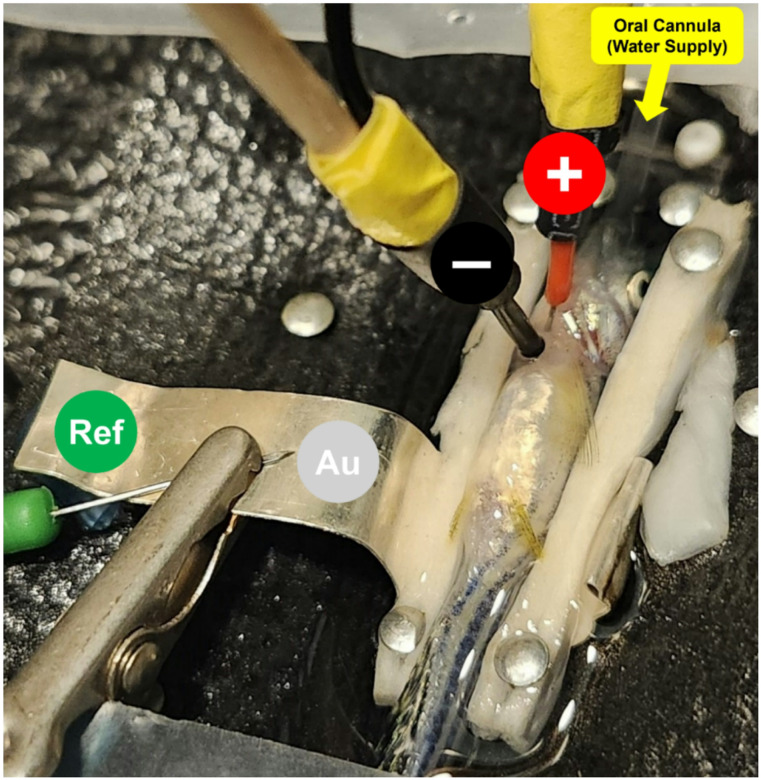
Single-lead ECG setup on orally cannulized adult zebrafish. Positive electrode (+, red), negative electrode (−,black), and ground electrode (Ref, green) contacting silver plating (Au, silver) are inserted subdermally, under the scales and ~1 mm deep into the chest musculature under anesthesia.

**Figure 2:**
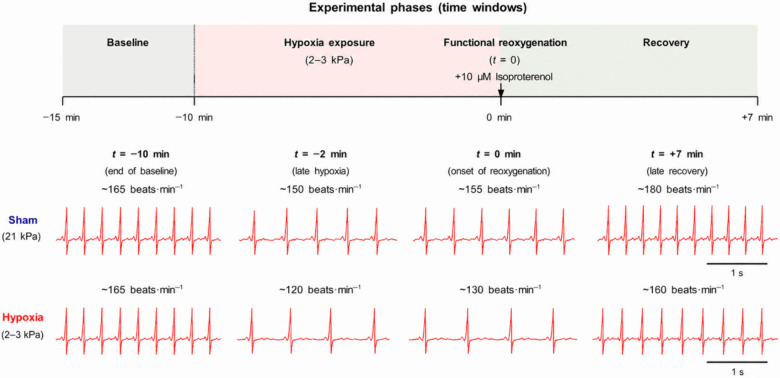
Representative single-lead ECG recordings sampled at indicated timestamps during experimental windows. Four representative ECG traces recorded at defined protocol timepoints illustrate cardiac activity throughout sham and hypoxia procedures. MS-222 was discontinued upon functional reoxygenation, and 10 μM isoproterenol was introduced at t = 0.

**Figure 3:**
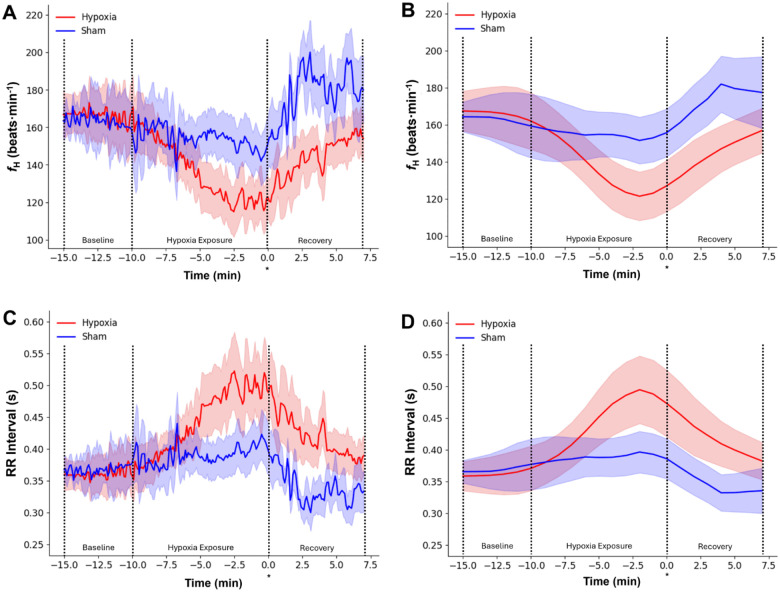
Mean *f*_*H*_ and RR interval responses to acute hypoxia and reoxygenation in adult zebrafish. (a) Mean heart rate (beats min^−^^1^±s.e.m.) for hypoxia (red, n = 8) and sham (blue, n = 7) groups aligned to each fish’s functional reoxygenation timepoint (t = 0). (b) LOESS-smoothed heart rate trajectories (shaded regions = ±s.e.m.) showing the continuous pattern of rate change across the −15 to +7 min experimental time course. (c) Mean RR interval (s ±s.e.m.) over the same time course. (d) LOESS-smoothed RR interval trajectories (shaded regions = ±s.e.m.). Time-series values were averaged into 25 s bins, and LOESS smoothing was applied (fraction = 0.3, degree = 1). Vertical dashed lines indicate boundaries between the baseline, hypoxia exposure and recovery phases, with functional reoxygenation at t = 0. The asterisk indicates the time of functional reoxygenation. LOESS-derived values were used for full-window and window-specific two-way ANOVAs.

**Figure 4:**
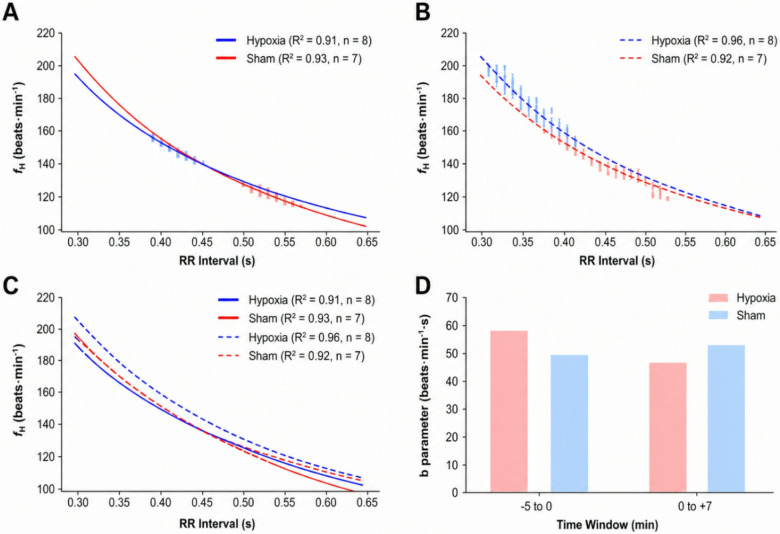
Non-linear regression analysis of the relationship between *f*_*H*_ and RR interval in adult zebrafish during hypoxia and reoxygenation. plotted as a function of RR interval for Hypoxia (red) and Sham (blue) groups across two time windows: −5 to 0 min **(a)** and 0 to +7 min **(b)**. Scatter points represent 5 s binned mean values with s.e.m., and lines indicate non-linear fits to the reciprocal model. Corresponding R^2^ values for each fit are shown in the legends. **(c)** Overlay of fitted curves from both time windows for each group, comparing −5 to 0 min (solid lines) and 0 to +7 min (dashed lines). **(d)** Estimated parameter values from the fitted model for each group and time window.

**Figure 5:**
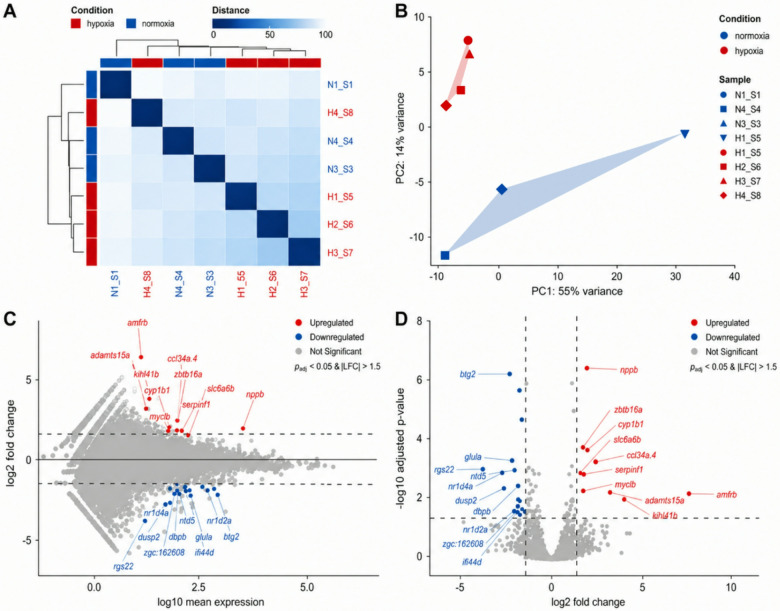
Adult zebrafish ventricular transcriptome responses 7 days after acute hypoxia exposure. **(a)** Hierarchically clustered sample-to-sample distance matrix of variance-stabilized (VST) expression profiles (hypoxia, n = 4, normoxia, n = 3). Darker cells indicate greater transcriptomic similarity. **(b)** Principal component analysis (PCA) of the 500 most variable genes showing sample clustering by condition; PC1 and PC2 explain 55% and 14% of variance, respectively. **(c)** Minus-average (MA) plot of transcript abundance versus log_2_ fold change relative to normoxia. Differentially expressed transcripts (DESeq2; p_adj_ < 0.05, |log_2_FC| > 1.5) are shown as upregulated (red), downregulated (blue), or nonsignificant (gray); dashed lines indicate fold-change thresholds. Top 10 up- and downregulated transcripts are labeled. **(d)** Volcano plot of differential transcript expression showing log_2_ fold change versus −log_10_ p_adj_, with the same significance criteria and transcript labeling as in **(c)**. Dashed lines indicate fold-change and significance thresholds.

Following cannulation and the onset of anesthesia, positive and negative electrodes were inserted at an initial angle of approximately 15°, guided beneath the scales until piercing the ventral skin. After penetrating the skin, each electrode was rotated upright to a final orientation perpendicular to the body (90°) and advanced ~ 1 mm into the ventral musculature by fine micromanipulator adjustments. The positive (red) electrode was placed at the ventral midline near the bulbus arteriosus, the negative (black) electrode was placed ~ 5–6 mm caudal to the positive electrode and slightly left of midline, and the reference (green) electrode was clamped to a strip of silver plating resting under the fish for consistent grounding (Fig. 1). Electrode positions were fine-tuned using the micromanipulators while observing the live ECG trace to maximize trace signal clarity. Note that, unlike ECGs made with typically 10 electrodes, the three-electrode configuration used in these experiments does not convey quantitative information regarding signal amplitude.

The ECG apparatus containing the adult fish was located inside a Faraday cage to minimize external electrical interference, while all electronic equipment and lighting remained outside the cage. Aluminum foil was draped over the sides and top of the cage to enhance electromagnetic shielding. ECG signals were amplified using an FE136 Animal Bio Amp and recorded via a PowerLab 8/35 data acquisition system (Model PL3508; ADInstruments, Colorado Springs, CO, USA) at 1–2 kHz using LabChart v8.1.28. A digital 5–50 Hz band-pass filter was applied during analysis to reduce low-frequency drift, motion artifacts, and high-frequency noise while preserving ECG wave morphology. Continuous ECG signal was captured throughout baseline normoxia, hypoxia, and reoxygenation phases.

### ECG Data Analysis

ECG waveform data were analyzed using the ECG Analysis Module in LabChart v8.1.28 (ADInstruments). Raw RR interval and heart rate *f*_*H*_ values were exported for all hypoxia and sham individuals. For each fish, time values were aligned such that *t* = 0 min corresponded to the onset of *functional* reoxygenation, i.e., the point at which pronounced *f*_*H*_ and RR interval rebound were readily apparent. RR interval and *f*_*H*_ values were averaged into 25 sec time bins to reduce variability. LOESS smoothing (fraction = 0.3; degree = 1) was then applied to generate continuous mean *f*_*H*_ and RR interval traces for both hypoxia and sham groups.

Non-linear regression was performed separately for the − 5 to 0 min and 0 to + 7 min windows of each treatment group as these intervals correspond to periods immediately before and after functional reoxygenation when changes in the relationship between *f*_*H*_ and RR interval are most pronounced. Mean ± s.e.m. values for *f*_*H*_ and RR interval were organized into 5 s time bins to reduce variability. The relationship between f and RR interval was modeled using a reciprocal function:

(1) $$f_{H}=\alpha +\frac{b}{RR}$$

where α represents the intercept (baseline offset in *f*_*H*_). Eq. (1) was used to fit curves for each group and time window. R^2^ values represented goodness of curve fit. The b parameter represents the reciprocal scaling constant that determines curvature and magnitude of the *f*_*H*_–RR interval relationship. Larger b values indicate a steeper reciprocal relationship, meaning *f*_*H*_ increases more sharply for a given decrease in RR interval.

#### Statistical Analyses of Cardiac Trace Data.

LOESS-smoothed *f*_*H*_ and RR interval values were analyzed by two-way ANOVA Type III given the unequal n values between the hypoxia and sham groups. The two factors compared were time (pre- vs. post-reoxygenation) and group (sham vs. hypoxia). ANOVAs were performed for the full − 5 to + 7 min window and separately for the − 5 to 0 min and 0 to + 7 min intervals to distinguish hypoxic suppression from post-reoxygenation recovery. Because LOESS smoothing of the cardiac trace data reduces noise and produces clearer trajectory lines, the ANOVA p-values represent differences in group-level trends over time rather than differences in individual-level variability. All analyses (LOESS, non-linear regression, and ANOVA) were performed using Matplotlib 3.8.2. and Statsmodel 0.14.0. Only individuals that survived the full hypoxia–reoxygenation sequence were included in the *f*_*H*_ and RR-interval analyses.

### Transcriptomics

Ventricular transcriptomes from hypoxia-exposed and normoxic control adult zebrafish were analyzed. Upon thawing, ventricles were immediately placed in TRI Reagent (Zymo Research, Irvine, CA, USA) and homogenized at room temperature by repeated shearing through an 18-gauge needle and syringe. RNA was then extracted using a Direct-zol RNA Miniprep Kit (Zymo Research) according to the manufacturer’s instructions. Following extraction, RNA quality was assessed using the Agilent TapeStation 4200 (Agilent, Inc., Santa Clara, CA, USA) with RNA ScreenTape assays. Samples with an RNA Integrity Number (RIN) ≤ 7 were excluded or re-extracted, where the RIN is a standardized metric of RNA integrity ranging from 1 (degraded) to 10 (intact).

Libraries were prepared from high-quality RNA using the Illumina Stranded mRNA Prep Kit (Illumina, Inc., San Diego, CA, USA) and sequenced on the Illumina NextSeq 500 platform using 150-cycle mid-output kits (paired-end, 2 × 75 bp).

Lane-level FASTQ files were concatenated per sample, and adapter trimming and quality filtering were performed using fastp v1.0.1 (minimum Phred score 20, minimum read length 36 bp) with automatic paired-end adapter detection. Post-trimming quality was assessed with FastQC v0.12.1. Trimmed reads were aligned to the zebrafish reference genome (GRCz12tu; GCF_049306965.1) using STAR v2.7.11b with the --quantMode GeneCounts option to generate gene-level read counts. The STAR genome index was built with --sjdbOverhang 74 to match the read length. Alignment rates for included samples ranged from 91.6% to 95.1% uniquely mapped reads. Gene-level reverse-stranded counts were extracted from STAR output (ReadsPerGene.out.tab, column 4) and assembled into a count matrix. One normoxia replicate (N2) was excluded due to library failure, yielding 28,888 reads compared to 5.9–17.9 million reads for the remaining samples. Differential expression analysis was performed using DESeq2 v1.46.0 in R v4.4.3, with normoxia set as the reference condition. Variance-stabilizing transformation was applied for sample-level quality control, including principal component analysis (PCA) and sample distance clustering heatmaps. Genes were considered differentially expressed at an adjusted p-value (p_adj_) (Benjamini–Hochberg procedure) < 0.05 and an absolute log_2_ fold change ≥ 1.5. Volcano plots and heatmaps were generated using ggplot2 v4.0.2 and the Python seaborn v0.13.2 and matplotlib v3.10.8 libraries. The Anthropic-developed large language model Claude Opus 4.6, accessed via Claude Code in Microsoft Visual Studio Code, was used for code implementation, refactoring, and debugging in a manner consistent with APS ethical policies; the authors take full responsibility for the content.

We compared our transcriptomic results with those of Marques et al.^25^, a microarray study of adult zebrafish hearts under hypoxic conditions to provide context for the acute hypoxia response.

Differentially expressed genes were extracted from their supplemental tables and compared against our 27 DEGs to identify shared genes and directionality of response. This comparison should be interpreted cautiously given fundamental differences between the two studies, including platform and gene coverage (Affymetrix microarray vs. RNA-seq), statistical thresholds (≥ 2-fold change with p ≤ 0.02 vs. Benjamini–Hochberg adjusted p < 0.05 with |log_2_FC| ≥ 1.5), and experimental design. Marques et al.^25^ exposed adult zebrafish to chronic constant hypoxia (10% air saturation) for three weeks and collected whole hearts during active hypoxia, whereas the present study exposed fish to acute hypoxia (pO_2_ = 2–3 kPa, up to 10 min) and collected ventricles seven days post-recovery under normoxic conditions.

## Figures and Tables

**Table 1:** Differentially expressed genes in adult zebrafish ventricles at seven days after acute hypoxia exposure.

Gene	Log_2_FC	P_adj_	Gene ID	Full Gene Name	Human Ortholog
*amfrb*	6.66	0.00714	ZDB-GENE-130530-643	autocrine motility factor receptor b	AMFR
*klhl41b*	3.83	0.013	ZDB-GENE-030131-9875	kelch-like family member 41b	KLHL41
*adamts15a*	3.16	0.00781	ZDB-GENE-060526-203	ADAM metallopeptidase with thrombospondin type 1 motif, 15a	ADAMTS15
*ccl34a.4*	2.39	0.000768	ZDB-GENE-070912-32	chemokine (C-C motif) ligand 34a, duplicate 4	CCL (teleost paralog)
*cyp1b1*	1.99	0.000297	ZDB-GENE-030902-1	cytochrome P450, family 1, subfamily B, polypeptide 1	CYP1B1
*nppb*	1.9	4.75E-07	ZDB-GENE-130530-642	natriuretic peptide B	NPPB
*serpinf1*	1.79	0.00197	ZDB-GENE-040912-2	serpin peptidase inhibitor, clade F (alpha-2 antiplasmin, pigment epithelium derived factor), member 1	SERPINF1
*zbtb16a*	1.78	0.000248	ZDB-GENE-030131-1989	zinc finger and BTB domain containing 16a	ZBTB16
*myclb*	1.75	0.00708	ZDB-GENE-030131-5561	MYCL proto-oncogene, bHLH transcription factor b	MYCL
*slc6a6b*	1.53	0.00184	ZDB-GENE-030131-3077	solute carrier family 6 member 6b	SLC6A6
*nupr1b*	−1.54	0.034	ZDB-GENE-030131-4653	nuclear protein 1b	NUPR1
*ucp3*	−1.7	0.028	ZDB-GENE-040426-1317	uncoupling protein 3	UCP3
*rnf207a*	−1.7	0.0000243	ZDB-GENE-120813-7	ring finger protein 207a	RNF207
*slc25a43*	−1.8	0.042	ZDB-GENE-030616-69	solute carrier family 25 member 43	SLC25A43
*ccng2*	−1.82	0.00000269	ZDB-GENE-021016-1	cyclin G2	CCNG2
*nr4a1*	−1.82	0.015	ZDB-GENE-040704-11	nuclear receptor subfamily 4, group A, member 1	NR4A1
*cdkn1a*	−1.92	0.014	ZDB-GENE-070705-7	cyclin dependent kinase inhibitor 1A	CDKN1A
*nr1d2a*	−1.93	0.024	ZDB-GENE-040504-1	nuclear receptor subfamily 1, group D, member 2a	NR1D2
*dbpb*	−1.94	0.0051	ZDB-GENE-100922-6	D site albumin promoter binding protein b	DBP
*ifi44d*	−1.95	0.034	ZDB-GENE-200128-2	interferon-induced protein 44d	IFI44/IFI44L family
*zgc:162608*	−2.13	0.034	ZDB-GENE-070410-86	zgc:162608	
*ntd5*	−2.13	0.00157	ZDB-GENE-031030-7	ntl-dependent gene 5	APOH
*btg2*	−2.19	8.72E-07	ZDB-GENE-000210-15	B-cell translocation gene 2	BTG2
*glula*	−2.25	0.000759	ZDB-GENE-030131-688	glutamate-ammonia ligase (glutamine synthase) a	GLUL
*dusp2*	−2.71	0.0062	ZDB-GENE-040801-188	dual specificity phosphatase 2	DUSP2
*nr1d4a*	−2.8	0.00197	ZDB-GENE-080403-5	nuclear receptor subfamily 1, group D, member 4a	NR1D1
*rgs22*	−3.84	0.00148	NCBI GeneID 101884083	regulator of G protein signaling 22	RGS22

Summary of all significantly regulated (down or up) genes identified by DESeq2 (p_adj_ < 0.05, |log_2_ fold change| ≥ 1.5). Gene symbols, full names, and ZDB-GENE identifiers are from ZFIN (zfin.org); human orthologs and functional annotations were cross-referenced with NCBI RefSeq for the GRCz12tu assembly (GCF_049306965.1).

## Data Availability

All transcriptomic data for this study are available in the NCBI Gene Expression Omnibus (GEO) under accession number GSE319817 at https://www.ncbi.nlm.nih.gov/geo/query/acc.cgi?acc=GSE319817. Reviewer access during peer review is available using the token: afahgakcdlwrlwl. Scripts used for transcriptomic data processing and analysis are available at https://github.com/rayhendricks/zebrafishHypoxiaPublication. All non-transcriptomic data are available upon request.
